# Latent space unsupervised semantic segmentation

**DOI:** 10.3389/fphys.2023.1151312

**Published:** 2023-04-25

**Authors:** Knut J. Strommen, Jim Tørresen, Ulysse Côté-Allard

**Affiliations:** ^1^ Department of Informatics, University of Oslo, Oslo, Norway; ^2^ RITMO, University of Oslo, Oslo, Norway

**Keywords:** multi-dimensional time series, semantic segmentation, unsupervised learning, change-point detection (CPD), biosignal processing, machine learning, autoencoder

## Abstract

The development of compact and energy-efficient wearable sensors has led to an increase in the availability of biosignals. To effectively and efficiently analyze continuously recorded and multidimensional time series at scale, the ability to perform meaningful unsupervised data segmentation is an auspicious target. A common way to achieve this is to identify change-points within the time series as the segmentation basis. However, traditional change-point detection algorithms often come with drawbacks, limiting their real-world applicability. Notably, they generally rely on the complete time series to be available and thus cannot be used for real-time applications. Another common limitation is that they poorly (or cannot) handle the segmentation of multidimensional time series. Consequently, the main contribution of this work is to propose a novel unsupervised segmentation algorithm for multidimensional time series named Latent Space Unsupervised Semantic Segmentation (LS-USS), which was designed to easily work with both online and batch data. Latent Space Unsupervised Semantic Segmentation addresses the challenge of multivariate change-point detection by utilizing an autoencoder to learn a 1-dimensional latent space on which change-point detection is then performed. To address the challenge of real-time time series segmentation, this work introduces the Local Threshold Extraction Algorithm (LTEA) and a “batch collapse” algorithm. The “batch collapse” algorithm enables Latent Space Unsupervised Semantic Segmentation to process streaming data by dividing it into manageable batches, while Local Threshold Extraction Algorithm is employed to detect change-points in the time series whenever the computed metric by Latent Space Unsupervised Semantic Segmentation exceeds a predefined threshold. By using these algorithms in combination, our approach is able to accurately segment time series data in real-time, making it well-suited for applications where timely detection of changes is critical. When evaluating Latent Space Unsupervised Semantic Segmentation on a variety of real-world datasets the Latent Space Unsupervised Semantic Segmentation systematically achieves equal or better performance than other state-of-the-art change-point detection algorithms it is compared to in both offline and real-time settings.

## 1 Introduction

The physiological processes occurring in the human body generate a plethora of biosignals (e.g., motion, muscle activity, biopotential) that can provide otherwise inaccessible insights into a person’s health and activity Demrozi et al. (2020), and may even be leveraged for the development of human-computer interfaces Campbell et al. (2020). Through the rise of the Internet of Things and the development of more compact and energy efficient sensors, wearable technologies can now provide continuous, non-intrusive and multimodal monitoring of these biosignals in real-time. However, the sheer amount of data generated by these devices make the processing and analysis of this information challenging and time-consuming to perform. As an example, a single 9-axis inertial measurement unit cadenced at a low sampling rate of 60 Hz generates around 2 million data-points every hour. Having to manually annotate these unlabelled data streams can thus rapidly become impractical. Another commonly occurring type of data are weakly labeled time series which include imprecise or inexact labels of when a change actually occurred. For both unlabeled and weakly labeled time series, the most useful information often lies in the precise location where a transition in the time series take place. Thus to make use of this data at scale, being able to identify these critical points in the time series in an unsupervised manner would be highly beneficial. Unsupervised change-point detection (CPD) algorithms attempt to identify these abrupt change in the data generating process Aminikhanghahi and Cook (2017).

Unfortunately, CPD algorithms also tend to suffer from limitations that reduce their suitability for real-world applications. Notably, they tend to make assumptions and require prior knowledge of the data which in practice implicitly or explicitly restrict them to a specific domain (as opposed to being domain agnostic) Aminikhanghahi and Cook (2017); Gharghabi et al. (2017); Lin et al. (2016). Another common limitation is that many CPD algorithms are defined only for offline applications (i.e., they require access to the full time series before performing the segmentation) Aminikhanghahi and Cook (2017); Truong et al. (2020). Further, for many real-world applications fast change-point detection are necessary to execute time-sensitive actions. Thus, algorithms that cannot handle online streaming data are ill-adapted for this type of reality, as they require a complete rerun every time a new data point is added. This problem is compounded by the fact that time series are often recorded over a long time and with a high sampling rate. Thus, algorithms with high computational complexity or poor scalability are also inappropriate for these types of real-world applications. Therefore, creating a domain-agnostic, hyperparameter-light unsupervised semantic segmentation algorithm that can handle online streaming data at scale is a highly desirable goal.

One state-of-the-art algorithm, which meets many of these requirements is Fast Low-cost Unipotent Semantic Segmentation Gharghabi et al. (2017) (FLUSS). FLUSS is a domain agnostic, scalable CPD algorithm, that can work with both online and offline data. The main hypothesis behind the development of FLUSS is that subsequences (small snippets of the time series data extracted around each time step in the time series) in similar segments are more similar than subsequences occurring after a change-point. FLUSS handles multidimensional data by implicitly calculating the likelihood of a change-point at each time step for each channel independently before taking the average likelihood over all the channels. However, some of the considered dimensions might not contain information that is helpful for the segmentation, or they might be heavily correlated. Thus, when taking the average across all the channels, these “not so useful” channels will dilute the information from the meaningful channels. The authors acknowledge this problem and recommend that when dealing with high dimensional data one should do a search or manually find the most useful subset of channels and remove the rest.

As time series data are often sampled from multiple sources and sensors, especially when working with wearable devices, it would be helpful to have an algorithm that can automatically extract the most useful information from high-dimensional time series data during segmentation. Consequently, the main contribution of this work is the introduction of Latent Space Unsupervised Semantic Segmentation (LS-USS). LS-USS is a domain-agnostic unsupervised semantic segmentation method that can handle both online streaming and offline multidimensional data with easy-to-tune hyperparameters. LS-USS is based on FLUSS, but differs in its approach by identifying similarities between lower-dimensional encodings of the multidimensional subsequences, obtained through the use of an autoencoder, instead of similarities between the subsequences themselves. The idea is that the dimensionality reduction learned by the autoencoder will reduce redundant and correlated information between channels, which in turn will improve time series segmentation.

This work is organized as follows; an overview of the related work is given in Section 2. Section 3 introduces the notions necessary for the description of the proposed algorithm in Section 4. The experiments are then presented in Section 5. Finally, the results and their associated discussion are covered in Section 6 and Section 7 respectively.

## 2 Related work

CPD algorithms can be divided into two categories depending on their reliance on labeled data: Supervised and Unsupervised CPD. Supervised CPD usually entails extracting subsequences over a sliding window where each subsequence is assigned a class or label. Most of the classifiers used in other machine learning tasks are also leveraged for supervised CPD such as naïve Bayes Reddy et al. (2010), Support Vector Machines (SVMs) Reddy et al. (2010), Gaussian Mixture Models (GMMs) Reddy et al. (2010), Decision Trees Reddy et al. (2010), Hidden Markov Models Cleland et al. (2014), and Neural Networks Ronneberger et al. (2015). When labels are available, the supervised methods are often the preferred solution. Unfortunately, labeling time series data can be prohibitively expensive (in terms of time, cost and/or human labor) making it an impractical solution for a wide variety of domains. Another challenge with supervised CPD is that they generally require training samples from all possible states (classes) of the signals beforehand and thus cannot easily adapt to novel behavior in the time series. Therefore, unsupervised CPD remains of great interest in many practical applications.

Most of the existing unsupervised CPD algorithms make statistical assumptions on the data (e.g., stationarity, independent and identically distributed) Kuncheva (2013); Adams and MacKay (2007); Malladi et al. (2013); Harchaoui et al. (2008); Rosenbaum (2005); Friedman and Rafsky (1979); Itoh and Kurths (2010), and/or require extensive tuning of model parameters as they rely on predefined parametric models Kawahara et al. (2007); Itoh and Kurths (2010); Yamanishi and Takeuchi (2002). As such, this type of algorithm can be cumbersome to apply on new domains and less robust to changes in the data over time. In contrast, LS-USS was designed to work using only minimal assumptions about the data and without requiring domain knowledge.

Another common limitation of existing CPD algorithms is that they require batched data to function and are thus ill-suited for real-time applications Aminikhanghahi and Cook (2017); Rakthanmanon et al. (2011); Kawahara et al. (2007); Itoh and Kurths (2010) such as detecting change-points in a patient’s vital signs Yang et al. (2006) or continuously monitoring the wear and tear of industrial robots Nentwich and Reinhart (2021). In contrast, LS-USS can be efficiently updated every time a new data point is added to the time series, allowing it to run in real-time.

A popular form of unsupervised CPD algorithms relies on clustering subsequences of the time series Yairi et al. (2001); Fu et al. (2001); Li et al. (1998). These methods cluster individual subsequences extracted from running a sliding window over the time series. The idea being that if two temporally close subsequences belong to different clusters, a change-point most likely exist between the two. However, Keogh et al. Keogh and Lin (2005) have shown that using subsequence clustering essentially produces cluster centers which tend towards a sinusoidal signal with random phases that average to the mean of the time series for any dataset used. In other words, the change-points detected are essentially random. To address this issue, Keogh et al. Keogh and Lin (2005) proposes to instead cluster the time series data points using *motifs*. In the context of data mining, motifs can be seen as fingerprints for time series data, as all non-random time series data produced by the same process are bound to contain some reoccurring patterns. Thus, the difference between clustering motifs and subsequence clustering is that in the former case clusters are made up of short individual time series, while in the latter case the clusters are derived from subsequences extracted from a sliding window. This means that cluster centers in motif clustering represent averages over motifs (i.e., similar patterns in data), rather than averages over all the data. Several methods have been proposed to extract motifs in time series data Mueen et al. (2009); Rakthanmanon et al. (2011); Bailey (2021); Yeh et al. (2016). In particular, *matrix profile* Yeh et al. (2016) is a recent approach to motif discovery based on efficient calculation of the self-similarity matrix. As matrix profiles are a core aspect of LS-USS, they will be presented in more detail in Section 3.1. In Rodrigues et al. (2022), the authors propose a novel segmentation method that uses a feature-based self-similarity matrix (SSM) to measure the pairwise distance between subsequences of a time series. Unlike previous works, such as Yeh et al. (2016) and our own work, where the SSM is based on the raw time series or a latent representation of it, this method selects features from the Time Series Feature Extraction Library (TSFEL) Barandas et al. (2020) to construct the SSM. The authors then employ three techniques, namely, novelty search, periodic search, and similarity profile analysis, to segment the time series based on the constructed SSM.

**FIGURE 1:**
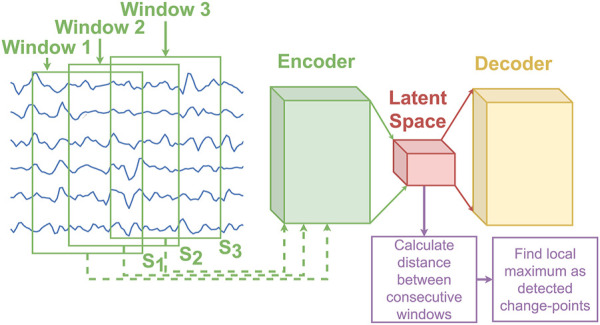
The diagram of the LFMD algorithm presented in Lee et al. (2018). The distance of the latent features are calculated between each adjacent window. The distance’s local maxima are selected as the change-points to be returned by the algorithm. Note that the overlap between window is an hyperparamter that has to be optimized for.

Many of the current states-of-the-art CPD algorithms Alippi et al. (2015); Gu et al. (2010); Kawahara and Sugiyama (2012); Kawahara et al. (2007), including FLUSS Gharghabi et al. (2017), were primarily designed for one-dimensional data. Unfortunately, this often limits their ability to effectively process multidimensional data Aminikhanghahi and Cook (2017), as they don’t effectively consider the varying levels of correlated information across dimensions, which can increase the complexity of finding change-points accurately. To alleviate this issue, CPD algorithms have been specifically designed for multidimensional data Qahtan et al. (2015); Kim et al. (2019); Lee et al. (2018); Sakurada and Yairi (2014); Zhou and Paffenroth (2017); Zhang et al. (2019). In Qahtan et al. (2015) principal component analysis (PCA) is first used to obtain a one-dimensional signal (using the principal component) before performing the segmentation. Kim et al. Kim et al. (2019) aims to segment out driving patterns from sensors on driving vehicles by leveraging word2vec Mikolov et al. (2013) to make an encoded representation of time series data consisting of both categorical and numerical information in varying scales. Autoencoders Tschannen et al. (2018) have also been employed in the domain of anomaly detection with great success Sakurada and Yairi (2014); Zhou and Paffenroth (2017); Zhang et al. (2019). The most common approach is to first train the autoencoder on time series data which does not contain that contains minimal or no anomalies. At inference time, the reconstruction error is employed as a measure of how likely the current time series contain an anomaly. The idea being that if the autoencoder cannot effectively reconstruct the input signal, it is likely because it is dissimilar from the training data. While this approach successfully finds change-points that are anomalies, it isn’t straightforward to adapt it for time series segmentation as the different segments are a natural part of the data. As such, it should be expected that an autoencoder trained on specific segments will also have a low reconstruction error when the data seen during inference belongs to any of the type of segments seen during training. Nevertheless, as shown in Lee et al. (2018), unsupervised segmentation can still be performed using an autoencoder while achieving state-of-the-art results by calculating a distance between consecutive windows in the latent space. This method referred to as Latent Feature Maximal Distance (LFMD), is illustrated in Figure 1. The central idea behind LFMD, using a learned latent space of an autoencoder as a way to efficiently characterize multidimensional data for CPD is also a core concept in LS-USS. As such, LFMD will be used in this work to better contextualize the performance of the proposed algorithm. Deep Time Series Embedding Clustering (DeTSEC) Ienco and Interdonato (2020) is another example of segmentation using autoencoders on high dimensional multivariate data. It has two stages: first, an attentive-gated autoencoder that creates a vector embedding representation of the time series; second, a clustering refinement stage that optimizes a loss function that balances reconstruction and clustering objectives. The final cluster assignment is obtained by applying the K-means clustering algorithm on the embeddings.

## 3 Preliminaries

The following section presents an overview of the fundamental building blocks used by LS-USS.

### 3.1 Matrix profiles

**FIGURE 2:**
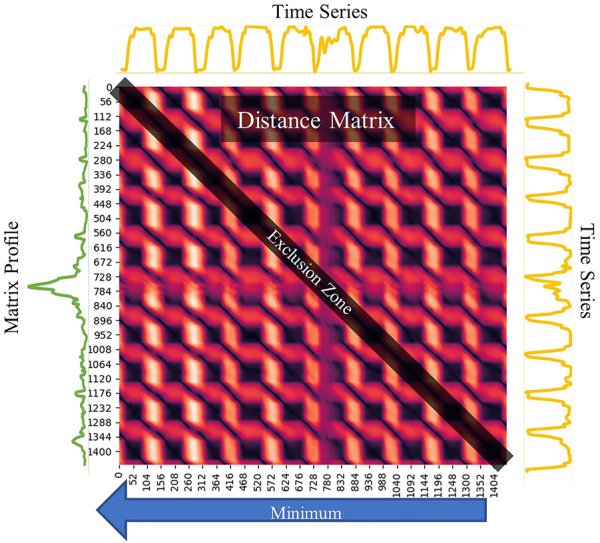
Plot depicting a time series and its corresponding distance matrix and matrix profile. The time series presented is an excerpt from the UCR GunPoint dataset from the UCR time series archive.

One of the core concepts behind LS-USS is the matrix profile Yeh et al. (2016), a data structure for time series that facilitates change-point detection and motif discovery. The matrix profile represents the distance between each motif and their most similar associated motif (excluding themselves). To calculate the matrix profile, one first has to find the set of all subsequence **A** from the time series $\vec{t_{A}}$ by utilizing a sliding window of length m∈N with a step size of 1 to extract all the possible subsequences of $\vec{t_{A}}$ . After this, a distance matrix is calculated by computing the z-normalized Euclidian distance between every subsequence in **A** with every other subsequence in **A**. Figure 2 shows what the resulting distance matrix looks like for the time series GunPoint from the UCR time series archive Dau et al. (2019). Each row in the distance matrix, referred to as the distance profile **D** depicts the distance from the subsequence at the current row index to every other subsequence in the time series. The distance profile **D** is calculated efficiently in $O\left(nlog\right.\left(n\right)$ , using a technique referred to as Mueen’s algorithm for similarity search (MASS) Rakthanmanon et al. (2012).

The matrix profile is obtained by calculating the Euclidean distances between every subsequence in **A** and its closest non-trivial neighbor in the same matrix. To create the matrix profile, the distance matrix is examined, and the minimum value in each row is extracted, representing the distance to the nearest neighboring subsequence. Note, however, that the most similar subsequence to a given subsequence will always be the subsequence itself (as they are identical). Similarly, subsequences that are extracted close in time will also be nearly identical. To avoid these trivial matches, an exclusion zone around each index is created by setting the values in these areas of **A** to infinity. As seen in Figure 2, this exclusion zone will be along the diagonal of the distance matrix.

**FIGURE 3:**
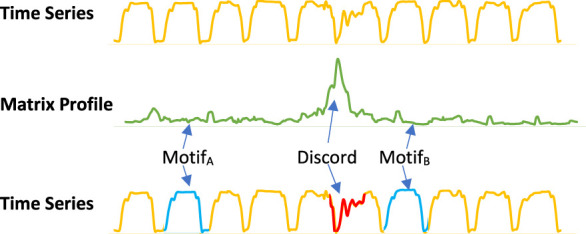
The distance matrix is a matrix constructed from all the distance profiles, which shows how similar each subsequence in the times series is to every other subsequence in the same time series. The matrix profile is derived by taking the minimum distance of each row. The original time series is shown in yellow and the matrix profile in green.

The matrix profile can be used to identify recurring patterns, known as motifs, and unique patterns, known as discords, in time series data. In the areas with relatively low values (i.e., high similarity), the subsequences in the original time series must have (at least one) relatively similar subsequence elsewhere in the data. These regions are reoccurring patterns which are classified as motifs and always come in pairs. In contrast, for areas with relatively high values (i.e., low similarity), the subsequence in the original time series must be a unique shape since it doesn’t match any other subsequence. These areas of discords can be considered anomalies in the data. Figure 3 shows an example of how motifs and discords can easily be identified using a matrix profile.

When calculating the matrix profile ${\vec{p}}_{A}$ , one can also extract the index of the nearest neighbor for each row in the distance matrix to make the matrix profile index ${\vec{i}}_{A}$ , which is defined as the vector containing the indices of the nearest non-trivial neighbor in **A** for every subsequence in **A**. The matrix profile index can thus be seen as a time series containing pointers to the nearest neighbors for each subsequence and will serve as the basis for the segmentation algorithm in LS-USS. The matrix profile can be efficiently calculated in *O*(*n* ^2^ log(*n*)) using the Scalable time series Anytime Matrix Profile (STAMP) Yeh et al. (2016) outlined in Algorithm 1.

Algorithm 1 The pseudo code for the algorithm Scalable Time Series Anytime Matrix Profile (STAMP) which is an efficient method for calculating the matrix profile.

**Input**:

$\vec{t_{A}}$ —*time series*
m—subsequence length
**Output**:

${\vec{p}}_{A},{\vec{i}}_{A}$ —*The incrementally updated matrix profile and the associated matrix profile index*
1: **procedure** STAMP( $\vec{t_{A}}$ *,m*)
2: ${\vec{p}}_{A}$ *= inf’s,* ${\vec{i}}_{A}$ *= zeros*
3: **A** ←*the all-subsequence set from* $\vec{t_{A}}$

4: **for** *idx = 0: length* $\left(\vec{t_{A}}\right)$ *-m* **do**
5: *D[idx] = MASS(* ***A*** *[idx],* $\vec{t_{A}}$ *) ⊳ MASS calculates the distance profile for the current subsequence*
6: ${\vec{p}}_{A}$ *[idx],* ${\vec{i}}_{A}$ *[idx] = ElementWiseMin(* ***D*** *[idx]) Save the minimum values in the distance profile and the corresponding index*
**return** ${\vec{p}}_{A},{\vec{i}}_{A}$

### 3.2 Fast Low-cost Unipotent Semantic Segmentation

Fast Low-cost Unipotent Semantic Segmentation (FLUSS) Gharghabi et al. (2017) is a segmentation algorithm that builds upon the matrix profile. This algorithm utilizes the matrix profile index $\left({\vec{i}}_{A}\right)$ , to segment time series data. The intuition behind FLUSS can be illustrated through this simple example:

When analyzing time series data collected from an inertial measurement unit worn by an individual who walks for a set period of time and then starts running, it is reasonable to expect that the walking subsequences will be highly similar to other walking subsequences, and the same for the running subsequences. Thus, for a given index in the time series $\vec{t}$ , the number of connections (arcs) crossing “over” that index would be low if it falls within an area of the time series where the activity is transitioning, whereas a high arc-count would be expected in areas with a clear homogeneous pattern. By counting the number of arc-crossings over each index, we can generate the Arc Curve (AC) which can be used to identify patterns and changes in the time series data.

The AC of a given time series $\vec{t}$ of length *n* will itself be a time series of length *n* containing only non-negative values. The value at the *i*th index in the AC specifies the number of arcs spatially crossing over location *i* in the original time series $\vec{t}$ Gharghabi et al. (2017).

**FIGURE 4:**
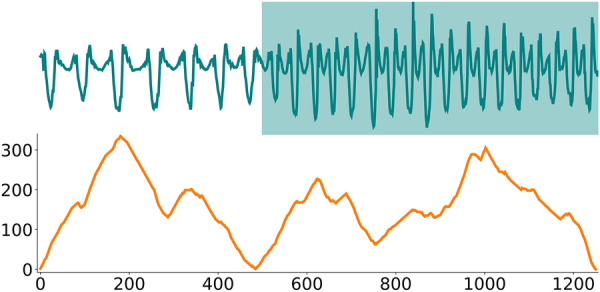
The top plots show a time series recorded from a gyroscope placed on a person’s arm Banos et al. (2014). During the recording, the person is initially walking but then starts jogging at around timestep 500. The bottom plot shows the corresponding arc curves. Importantly, the arc counts are low around timestep 500, corresponding to the time series true change-point. However, the arc counts also decrease towards the beginning and end of the time series, which corresponds to a construction artifact that has to be corrected for.

**FIGURE 5:**
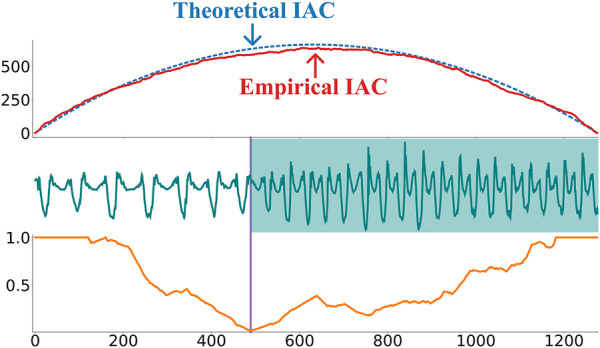
The top plot shows the empirical vs. theoretical idealized arc curve (IAC). The bottom plot shows the corrected arc curve (CAC) computed on the walking-jogging time series from Banos et al. (2014). As shown by the purple vertical line, the minimum value on the CAC can be used to identify this time series change-point.

Figure 4 shows that the AC is close to zero at the transition between the segments. However, by definition, the arc count will naturally be lower closer to the edges until it becomes zero as no arcs can cross the borders of the time series. To compensate for this, the authors in Gharghabi et al. (2017) divide the AC with an inverted parabola with a height equal to half the length of the time series. This parabola is called the idealized arc curve (IAC) and is what the AC would look like for a time series with no structure, where all arc curves would just point to random locations. The empirical and theoretical IAC is depicted in the top plot in Figure 5. Dividing the AC with the IAC will normalize the time series between 0 and 1 and solve the edge effects. The resulting vector is known as the Corrected Arc Curve (CAC). A min function is also applied to ensure that the CAC is between 0 and 1, even in the unlikely event that AC > IAC.

#### 3.2.1 Regime Extraction Algorithm

Low values in the CAC are used to identify potential change-points. In Gharghabi et al. (2017), change-points are then selected using the Regime Extraction Algorithm (REA). REA search for the *k* lowest “valley” points in the CAC. However, if the point at time *t* is the lowest point on the CAC, the points at time *t* − 1 and *t* + 1 are likely the second and third-lowest point. To avoid all the change-points ending up in one “valley”, an exclusion zone is set around the already detected “valley” points, the width of which is a hyperparameter. Note that REA only works when the number of change-points is known beforehand. Unfortunately such a requirement can in practice be hard/impossible to fulfill for a wide variety of applications. The pseudo-code for REA is outlined in Algorithm 2.

Algorithm 2 The pseudo code for the Regime Extraction Algorithm (REA) which is used for extracting change-points based on the CAC and number of change-points.

**Input**:
CAC—a Corrected Arc Curve
numRegimes—number of regime changes
NW—Subsequence size
**Output**:
locRegimes—the location of the change-points
1: **procedure** REA(*CAC,numRegimes, NW*)
2: *locRegimes = empty array of length numRegimes*
3: **for** *idx = 0: numRegimes* **do**
4: *locRegimes[i] = indexOf(*min*(CAC))*
5: *Set exclusion zone of 5* × *NW around locRegimes[i] ⊳ To prevent matches to “self”*
**return** *locRegimes*

#### 3.2.2 Fast Low-cost Online Semantic Segmentation

**FIGURE 6:**
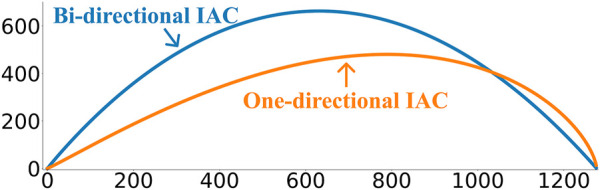
The bi-directional idealized arc curve (IAC) vs the one-directional IAC.

Adding a new point to the CAC takes only O(*n* log(*n*)). However, removing the oldest point takes O(*n* ^2^) as every subsequences could point to the ejected point, thus requiring the whole matrix profile to be updated. As such, while FLUSS can easily be run on offline datasets, it doesn’t scale well in the context of streaming data. To solve this issue, the authors in Gharghabi et al. (2017) propose an online version of FLUSS, referred to as Fast Low-cost Online Semantic Segmentation (FLOSS). FLOSS addresses the online streaming issue by explicitly forcing each arc to only point towards a newer data point. Consequently, as no arc can point to an older point, ejecting the oldest point in the currently considered time series can now be done in O(1). Thus, maintaining the one-directional CAC_1*D*_ can be done in O(*nlog*(*n*)). It should be noted however that in the case of the CAC_1*D*_, the IAC will now be skewed to the right as the rightmost part of the CAC_1*D*_ will have a higher chance of arc crossings (see Figure 6).

FLOSS in conjunction with the one-directional CAC thus enables online streaming using the matrix profile. However, it should be expected that FLOSS will perform worse than FLUSS due to the directional constraints imposed on the arcs.

#### 3.2.3 Locality—Temporal constraint

In some time series datasets, the same activities or events can arise multiple times in a disjointed manner (e.g., a time series representing the electrocardiogram of a person laying down and standing up multiple times in succession). In these cases, the CAC is not a good indicator of a regime change as the arcs for a given event-type (e.g., laying down) would have practically the same likelihood of pointing to subsequences in the segment they are occurring in than they would have in pointing to subsequences from other segments of the same event-type. This challenge can be addressed by using a temporal constraint (TC) that limits how far in time an arc can point, thus forcing each index in the CAC to only consider a local area around itself. While this adds a hyperparameter (length of TC) to the algorithm, it also reduces the computational time of FLUSS. If considered, this hyperparameter is suggested in Gharghabi et al. (2017) to be set to circa maximum expected segment length, allowing for the use of domain knowledge to the algorithm if available. Furthermore, as pointed out in Gharghabi et al. (2017), this hyperparameter isn’t very sensitive and thus only requires a rough idea of the order of the temporal scale at which the change-points occurs.

### 3.3 Autoencoder

Autoencoders Tschannen et al. (2018) are a type of neural network architecture designed to learn in an unsupervised manner a mapping from the high-dimensional input space to a lower-dimensional feature space (latent space) while preserving the salient information of the original observations. This mapping is learned by minimizing the distance between the original input and its corresponding reconstruction from the latent space.

More precisely, define X as the input space and consider a probability measure *p* on X . The objective of an autoencoder is then to learn two functions *ψ* and *ϕ*, such that:

ψ:X→Z (1)

ϕ:Z→X (2)

$$\psi ,\phi =\underset{\psi ,\phi }{\arg\;\min}E_{x∼p}\left[\left\|x-\left(\phi ◦\psi \right)\left(x\right)\right\|\right]$$ (3)

Where Z is the latent feature space that serves as an information bottleneck to the original feature space.

The input of the autoencoder is the time series partitioned into windows of length *w*. Note that these subsequences can be defined with an overlap. The detailed autoencoder architectures employed in this work will be presented in Section 4.

## 4 Materials and methods

LS-USS proposes to perform unsupervised segmentation by leveraging an autoencoder to learn a meaningful latent feature space which the corrected arc curve can be computed from. This section details the proposed algorithms and contributions of this work.

### 4.1 Autoencoder implementation

For comparison’s sake, the fully connected network architecture used by LFMD Lee et al. (2018) is employed in this work. Further, a simple convolutional network is also considered to evaluate if modeling the input’s temporal characteristics can help find a better latent representation than the fully connected version.

#### 4.1.1 Fully connected model

Following the autoencoder implementation Lee et al. (2018), the fully reproduced connected model uses two hidden layers for both the encoder and decoder, transposed weights for the decoder, and the sigmoid function as its activation function. For the decode encoder, each hidden layer is half the size of the previous layer and the opposite for the decoder layer (the initial size of which will depend on the input/subsequence size). Adam Kingma and Ba (2017) is employed to optimize the network’s weights using the mean square error as the loss function. Finally, the dimension of the latent space is defined so that the ratio between the feature representation and the input $\frac{\dim\left(x\right)}{\dim\left(z\right)}$ is 0.1. Where x∈X and z∈Z .

#### 4.1.2 Convolutional model

The architecture employed for the convolutional model is presented in Table 1. The model is trained in the same way as the fully connected model using mean square error as loss function and Adam as optimizer. The feature size, which is now defined as the ratio $\left(\frac{\dim\left(z\right)}{NC\times NW}\right)$ , is set to $\frac{1}{6}$ .

**TABLE 1:** Overview of the architecture used for the convolutional network version of the autoencoder. NC is the original number of channels from the input data, while NW represents the sub-sequence length.

Layer	Type	Input length	Output length	Input width	Output width	Kernel size	Stride	Padding	Activation
Hidden Layer 1 Encoder	Convolution	NW	NW/2	NC	2xNC	3	2	1	ReLU
Hidden Layer 2 Encoder	Convolution	NW/2	NW/4	2xNC	4xNC	3	2	1	ReLU
Reshape Encoder	Reshaping	NW/4	NW	4xNC	1	—	—	—	—
Hidden Layer 3 Encoder	Fully Connected	NW	NW/2	1	1	—	—	—	ReLU
Hidden Layer 4 Encoder	Fully Connected	NW/2	NW/4	1	1	—	—	—	ReLU
Hidden Layer 5 Encoder	Fully Connected	NW/4	NW/6	1	1	—	—	—	ReLU
Hidden Layer 1 Decoder	Transposed Hidden Layer 5 Encoder	NW/6	NW/4	1	1	—	—	—	ReLU
Hidden Layer 1 Decoder	Transposed Hidden Layer 4 Encoder	NW/4	NW/2	1	1	—	—	—	ReLU
Hidden Layer 2 Decoder	Transposed Hidden Layer 3 Encoder	NW/2	NW	1	1	—	—	—	ReLU
Reshape Decoder	Reshaping	NW	NW/4	1	4xNC	—	—	—	—
Hidden Layer 1 Encoder	Transposed Convolution	NW/4	NW/2	4xNC	2xNC	3	1	1	ReLU
Hidden Layer 1 Encoder	Transposed Convolution	NW/2	NW	2xNC	NC	3	2	1	Linear

### 4.2 Latent space matrix profile

As previously stated, LS-USS leverages an autoencoder as a way to learn a meaningful representation of a multidimensional time series. Because the time series is first segmented into windows with each window going through the autoencoder, the resulting representation of the time series in the latent space is not a continuous time series. Unfortunately, this also means that the matrix profile cannot be computed from this latent space using the STAMP algorithm as presented in Section 3.1 as doing so is only possible if a sliding window can be applied to the time series itself. Thus, this work introduces the Latent Space Matrix Profile (LSMP) which, as the name implies, is the matrix profile computed directly on the latent representation. The LSMP is defined by the same logic used for calculating the regular matrix profile (see Section 3.1). The difference being the use of latent representations instead of subsequences. By exploiting the fact that each distance calculation can be done independently, it is possible to implement the computation of the Euclidean distance between pairs of vectors to run in parallel (e.g., GPU). The pseudo-code to compute the LSMP is presented in Algorithm 3.

Algorithm 3 The pseudo code for calculating the Latent Space Matrix Profile (LSMP) which is a representation of the matrix profile that is based on the latent representations of the original time series.

**Input**:

$\vec{t_{A}}$ —*time series*
m—subsequence length
**Output**:

${\vec{p}}_{F}$ *,* ${\vec{i}}_{F}$ —*The incrementally updated latent space matrix profile and the associated latent space matrix profile index*
1: **procedure** LSMP( $\vec{t_{A}}$ *,m*)
2: ${\vec{p}}_{F}$ *= inf’s,* ${\vec{i}}_{F}$ *= zeros*
3: *F* ←*the latent all-subsequence set from* $\vec{t_{A}}$

4: **for** *idx = 0: length* $\left(\vec{t_{A}}\right)$ *-m* **do**
5: *D[idx] = CDIST(A[idx],* $\vec{t_{A}}$ *) ⊳ CDIST calculates the distance profile for the current subsequence.*
6: ${\vec{p}}_{F}$ *[idx],* ${\vec{i}}_{F}$ *[idx] = ElementWiseMin(D[idx]) Save the minimum values in the distance profile and the corresponding index*
**return** ${\vec{p}}_{F},{\vec{i}}_{F}$

**FIGURE 7:**
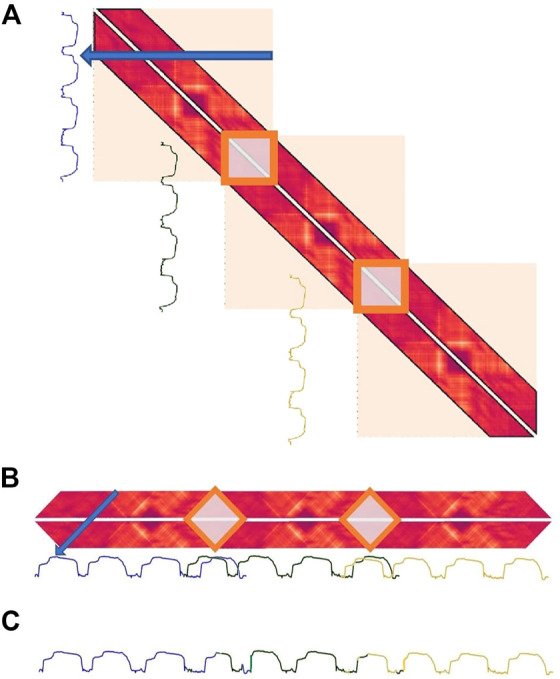
The top part of **(A)** (blue arrow) depicts the batched collapse of the temporally constrained latent distance matrix into the LSMP. **(A)** as a whole shows the temporarily constrained matrix profile of a time series of length 10,400. **(B)** shows the three batched matrix profiles calculated where they overlap with each other. **(C)** shows the full matrix profile after taking the minimum value in the overlapping area.

Similarly to FLUSS, performing time series segmentation based on the LSMP and CAC is only meaningful when the same segment-type isn’t repeated other places in the time series (see Section 3.2.3). Therefore, a TC is also applied when computing the CAC from the LSMP. Adding this TC also comes with the added benefit that only the distances between the latent features located inside the temporal constraint need to be calculated. The top of figure 7-A depicts the collapse of the temporally constrained latent distance matrix into the LSMP.

A memory problem arises for long time series as it is necessary to save 2*TC data points per time step to make the temporally constrained distance profile, which can rapidly become intractable. To address this memory issue, instead of considering the full distance matrix (constrained by TC), this work proposes using an overlapping window on the time series where a distance matrix will be computed for each window. A matrix profile for each distance matrix is then computed. Finally, the different matrix profiles are merged together by taking the minimum value (and the corresponding index) over the overlapping area of each matrix profile. This operation is referred to as the batched collapse of the matrix profile, a depiction of which is shown in Figure 7 (the pseudo-code is also provided in Algorithm 4).

Algorithm 4 The pseudo code for Batched Collapse Algorithm which is a method of calculating parts of the LSMP (with temporal constraints) for a given time series without the need to calculate the full distance profile.

1: **procedure** (*t*_lim_)
2: *D* ← *The distance matrix, calculated from timestamp 0 to* *t*_lim_
3: ${\vec{p}}_{F}\;\leftarrow$ *The matrix profile obtained by collapsing* *D*
4: *t*_lim  _*curr*_ ← *t*_lim_
5: **while** *t*_lim_ < *len*( $\vec{t_{A}}$ ) **do**
6: *t*_lim  _*prev*_ ← *t*_lim  _*curr*_
7: *t*_lim  _*curr*_ ← *t*_lim  _*curr*_ + *t*_lim_
8: *D*_*new*_ ← *Calculate distance matrix from* *t*_lim  _*prev*_ *-* *TC* × 2 − 1 *to* *t*_lim  _*curr*_
9: Collapse *D*_*new*_ to get the matrix profile ${\vec{p}}_{Fnew}$

10: ${\vec{p}}_{Fmerged}\;\leftarrow$ *Merge* ${\vec{p}}_{F}$ *with* ${\vec{p}}_{Fnew}$ *⊳ keep the minimum value between* ${\vec{p}}_{F}$ *and* ${\vec{p}}_{Fnew}$ *where the two vectors overlap in time*
11: ${\vec{p}}_{F}\;\leftarrow \;{\vec{p}}_{Fmerged}$

**return** ${\vec{p}}_{F}$

As seen in the Batched collapse algorithm, collapsing the matrix profile before processing the entire time series comes with the cost of recalculating the last (TC*2–1) time steps of the matrix profile. This recalculation is necessary as the first time steps in the new matrix profile can point back to the old ones (see Figure 7).

The “batch collapse” algorithm allows for the adaptation of LS-USS to an online setting by accumulating a batch of data before appending it to the matrix profile. This approach enables the algorithm to detect change-points with a minimum of *ϵ* data points, thus operating in *ϵ* real-time. However, it is important to note that this method incurs a trade-off between batch size and computation time, as the recalculation of *TC* × 2 − 1 time steps is required each time a batch is added. A completely online algorithm would be 1 real-time.

**FIGURE 8:**
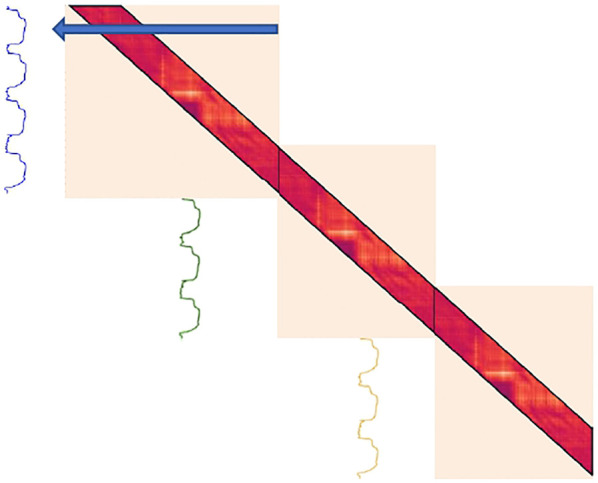
Online version of LSMP on a time series of length 10,400. No recalculation is needed when updating the distance matrix.

In section 3.2.2, FLOSS was made to work online by forcing arcs to only point forwards in time. This trick can also be borrowed to update the LSMP in real-time. If one only looks for the nearest-neighbor forward in time, no subsequence can have a nearest-neighbor in the previous LSMP. As such, the requirement of recalculating TC*2-1 time steps at each new batch also disappears (see Figure 8). As with FLOSS, it should be expected that using only forward-pointing arcs will have a negative impact on the algorithm’s performance.

### 4.3 Latent space unsupervised semantic segmentation (LS-USS)

LS-USS uses an autoencoder to encode the multidimensional all subsequences set **A** into the one-dimensional latent all subsequence set **F**. **F** is then used to compute the LSMP ${\vec{P}}_{F}$ and the corresponding LSMP index vector ${\vec{I}}_{F}$ as described in the previous section. The indices ${\vec{I}}_{F}$ are then used to make the CAC, which is the graph that contains the number of arc crossings at each time step. Like in FLUSS, few arc crossings over a particular data point indicates a high likelihood of a change-point at that time step, and a high number of arc crossings indicates a low likelihood for a change-point.

LS-USS online is similar to LS-USS, except that it uses the version of the LSMP that works online by only considering right-pointing arcs. Doing this makes it possible to update ${\vec{I}}_{F}$ without recomputing the distance matrix for the last (*TC* × 2 − 1) time steps. As mentioned, the regular LSMP can also be *ϵ* real-time by accumulating a batch of data before adding it to the end of the ${\vec{P}}_{F}$ , making the regular LS-USS *ϵ* real-time.

**FIGURE 9:**
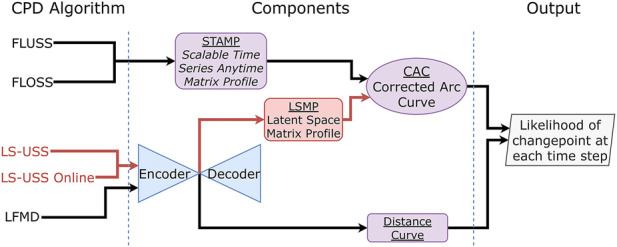
Overview of the LS-USS and LS-USS online algorithms compared to the other considered algorithms.

An overview of the relation between LS-USS, LS-USS online, FLUSS, FLOSS and LFMD is shown in Figure 9. The diagram shows that the LS-USS algorithms utilizes the same autoencoder as LFMD to learn a latent representation of a multidimensional time series. They also use the concept of matrix profiles from FLUSS/FLOSS (although it is here computed from latent features of the original data). The exact same algorithm is used to calculate the CAC for the FLUSS/FLOSS and the LS-USS algorithms.

### 4.4 Local Regime Extraction Algorithm

REA was presented as an algorithm for extracting segments based on the k-lowest “valley” points of the CAC. For most long time series data the change-points are distributed relatively evenly in time. In these cases, when extracting change-points, local minimums are often more meaningful than the global minimums. The REA algorithm will always pick the global k-lowest “valley” points on the CAC instead of locating where the CAC is at its lowest compared to the local area around it. To address this issue, this work introduces the Local Regime Extraction Algorithm (LREA). The method scales each point in the CAC to zero mean and unit variance based on the local mean and standard deviation calculated over a rolling window (as shown in Eq. 4). After the scaling, the same procedure used in the REA algorithm extracts the lowest k “valley points” from the scaled CAC.

$$CAC_{\text{scaled }}=\frac{CAC-\mu_{\text{rolling }}}{\sigma_{\text{rolling }}}$$ (4)

Note that by increasing the window size, the extraction threshold will be increasingly global and thus tend towards REA.

### 4.5 Local Threshold Extraction Algorithm

For comparisons between the offline CPD algorithms in this work, the algorithms REA and LREA are used. However, these algorithms require information about the number of segments, which is often unavailable in practice. To address this, this work introduces the Local Threshold Extraction Algorithm (LTEA). As the name suggests, this algorithm works by scaling the CAC before doing threshold-based change-point extraction.

The same CAC scaling used in the LREA algorithm is first applied to LTEA. Note that in the case where the algorithm is applied on an online (streaming) time series, the rolling statistics can naturally be computed only on prior data (which is the main difference with offline dataset).

In LTEA, the scaled CAC-values over a given threshold are disregarded by being set to a value of one. When the scaled CAC is standardized to zero mean and unit variance, a good threshold value was found to be around minus one (one standard deviation).

**FIGURE 10:**
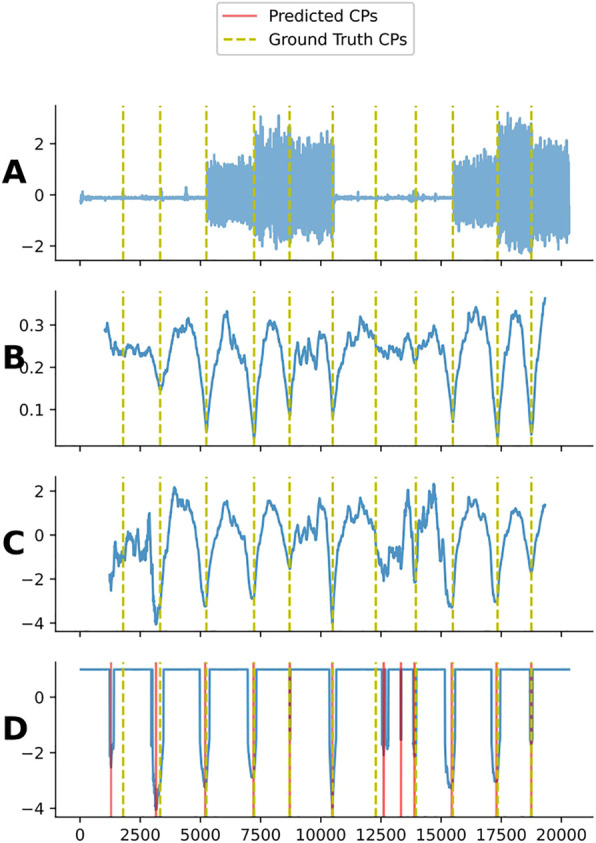
Plot **(A)** shows one channel from subject 4 in the UCI dataset. Plot **(B)** shows the regular CAC for subject 4. Plot **(C)** shows the scaled CAC. Plot **(D)** shows the thresholded CAC and the predicted change-points when using LTEA on this CAC. Note that the LTEA algorithm does not have prior knowledge of the number of change-points present in a given time series. As a result, it may detect more or less change-points than are actually present. In this example, an additional change-point, not present in the ground truth, was detected at an approximate index of 13,500.

After thresholding the CAC, the local minimum obtained are identified as the change-point location. An exclusion zone like the one used in LREA and REA is also applied to avoid trivial matches caused by valleys close in time. The pseudo-code to compute LTEA is presented in Algorithm 5 and an example of the application of LTEA is shown in Figure 10.

Algorithm 5 The pseudo code for the Local Threshold Extraction Algorithm (LTEA) which is used to extract change-points in an online fashion by scaling a CAC based on its local statistics and thresholding the resulting scaled CAC.

**Input**:
CAC–a Corrected Arc Curve or Distance Curve
localWindowSize–The size of the window used to normalize the CAC
*threshold–Values of the* *CAC*_*scaled*_ *above this threshold will be set to 1. A good default value is to set this to -1.*
**Output**:
locRegimes–the location of the change-points
1: **procedure** LTEA(*CAC, localWindowSize, threshold = -1.*)
2: *CAC*_*scaled*_ ← *empty array with same length as the CAC*
3: **for** i = 0:*length*(*CAC*_*scaled*_) **do**
4: *localWindow* ← *CAC[idx-(localWindowSize): idx+(localWindowSize)]*
5: *μ*_*rolling*_ ← *mean(localWidow)*
6: *σ*_*rolling*_ ← *std(localWindow)*
7: *CAC*_*scaled*_ *[i]* ← *(CAC[i] -* *μ*_*rolling*_ *)/* *σ*_*rolling*_
8: **if** *CAC*_*scaled*_ *[i]* > *threshold* **then**
9: *CAC*_*scaled*_ *[i]* ← *1* valleys ← List of sequences in *CAC*_*scaled*_, where a sequence corresponds to all the consecutive points with a value different than 1 in the *CAC*_*scaled*_
10: **for** *valley in valleys* **do**
11: *locRegimes[i]* ← *indexOf(*min*(valley))*
**return** locRegimes

## 5 Experiments

### 5.1 Datasets

To benchmark the performance of LS-USS (and LS-USS Online) against FLUSS, FLOSS and LFMD, five biosignal-based datasets are considered.

#### 5.1.1 UCI human activity recognition

The UCI Human Activity Recognition Using Smartphones Dataset Anguita et al. (2013) contains 30 volunteers between the ages of 19 and 48 who performed six activities wearing a smartphone on the waist. The activities were walking, walking up stairs, walking down stairs, sitting, standing, and laying down. The collected data came from the 3-axial accelerometer and 3-axial gyroscope located in a Samsung Galaxy S2 and were sampled at 50 Hz. The raw acceleration data signals have three main components: body movement, gravity, and noise. The sensor data was filtered using a median filter and a 3rd order low pass Butterworth filter with a corner frequency of 20 Hz for noise removal. A low pass Butterworth filter, with a corner frequency of 0.3 Hz, was used to separate the body movement and gravity signals. The reason for choosing to use a 0.3 Hz cut-off frequency is that gravitational forces are assumed only to have low-frequency components. This work will use the data from the gyroscope, accelerometer, and the filtered body movement from the accelerometer. As all these signals are sampled in all three spatial dimensions, the dataset contains nine channels in total. Nine subjects constitute the training set, five subjects are used as a validation set, while the remaining 16 subjects are picked for the test set. The labeled change-point detection is available in this dataset with the goal of detecting when the participant starts a new activity. This dataset will be referred to as the UCI dataset.

#### 5.1.2 EMG-based long-term 3DC dataset

The EMG-Based Long-Term 3DC Dataset Côté-Allard et al. (2021) contains data from 22 able-bodied participants who performed eleven hand gestures while recording their forearm’s muscle activity over a period of 21 days. The recording sessions took place every ∼7 day. The goal for the unsupervised segmentation is to detect when a participant transitions towards a new gesture. This is of particular interest for myoelectric-based control as being able to detect such transitions accurately in real-time would improve the performance of previously proposed self-learning classifiers Côté-Allard et al. (2020). The armband used when recording this dataset is the 3DC Armband Côté-Allard et al. (2019). The 3DC is a ten-channel, dry electrode 3D printed EMG band with a sampling rate of 1,000 Hz per channel. While the armband also features a 9-axis Magnetic, Angular Rate, and Gravity (MARG) sensor, only the 10 EMG channels are considered in this work. The dataset is divided into training sessions and evaluation sessions. In the training session, participants were asked to hold each of the 11 gestures for 5 s. The transitions between gestures weren’t recorded, yielding a discontinuous time series. This procedure was repeated four times by each participant on every recording day. For the evaluation session, participants were randomly asked to hold a total of 42 gestures. The requested gestures were selected at random every 5 s (the time series associated with an evaluation session thus lasted 210 s). Each participant recorded a minimum of 6 evaluation sessions (twice per recording session). Importantly, for the evaluation session, the transition between each gesture was recorded, yielding a continuous time series.

For this work, two datasets were derived from the Long-Term 3DC Dataset: The EMG Artificial Dataset and the EMG Dataset.

The EMG Artificial Dataset was created from the original training sessions by concatenating these gesture recordings together to form a continuous time series for each participant. The artificial training set consists of training sessions recorded from ten participants. The validation set consists of 15 time series made by gestures from four participants, while the test sets includes 30 time series from eight participants. Note that no participant is included in more than one set to avoid data leakage.

The EMG Dataset was constructed using data from the evaluation sessions of the Long-Term 3DC Dataset. Six participants’ data were used to create the training set, five participant’s data were used to form the validation set, and the remaining nine participant’s data were used to comprise the test set. Similarly to the EMG Artificial Dataset, no participant was included in more than one set.

#### 5.1.3 Expressive motion with dancers

The dataset Expressive motion with dancers St-Onge (2018) is made in collaboration with professional dancers performing different dance moves that correspond to three emotional states. The dancers were wearing two Myo armbands, one on the calf and one on the forearm. The Myo is a low-cost eight-channel consumer-grade, dry-electrode EMG armband that also integrates a nine degree of freedom IMU (inertial measurement unit). Data from the two IMU sensors are represented by yaw, pitch roll coordinates and are sampled at 50 Hz. The dancer was instructed to develop three choreographic moods differentiated by the emotion they subjectively represented for the performer. To generate training data, the dancer repeated each sequence of moods for around 20 s. In addition to this, the dancers also created dance performances using the same lexicon of sequences used when building the training set. The dataset contains data from 27 participants.

Here, the training dataset is used as the training set, while the dance performances from 9 participants are used as the validation set, and the remaining 18 are used for testing.

An additional artificial dataset is made from the original training data. The new training set includes raw data from 12 participants, while the validation and test sets have been made by randomly concatenating ten to seventeen choreographic moods from remaining six and nine participants, respectively. The validation set consists of 20 artificial time series, while the test set consists of 50.

The two datasets that are based on data from Expressive motion with dancers will hereafter be referred to as Dance and Dance Artificial.

### 5.2 Hyperparameter selection

The segmentation algorithms are divided into two categories: online and offline.

Even if they are not fully online, LS-USS, FLUSS and LFMD are also included in the online category as they can be made to work *ɛ* real-time when using a temporal constraint. The hyperparameter optimization is done using grid search by leveraging the previously described training and validation sets for the five differnt datasets considered in this work. The step size, used in LFMD, decides the amount of overlap between subsequences. For the segmentation algorithms that use an autoencoder (LS-USS and LFMD), the fully connected and convolutional model is included in the hyperparameter search. For the offline algorithms the extraction algorithms REA and LREA are included in the hyperparameter search. Four data scaling techniques are applied to the data for comparing the segmentation algorithms on the different datasets. These methods use the implementation provided in scikit-learn Pedregosa et al. (2011) and are widely used for machine learning and data mining tasks. The scaling is done independently over all channels, and the statistics used for scaling the data are computed using only the training data. The choice of scaling method is also incorporated into the hyperparameter search.

To summarize, FLUSS and LS-USS (and their online counterparts) require us to specify the subsequence size (NW) and the Temporal Constraint (TC), while LFMD requires us to specify subsequence size (NW) and a step size. LS-USS and LFMD also have the option between two different autoencoder architectures.

The mean distance between the change-points from the training set is calculated for use as the local window size for scaling the CAC when using the LREA and LTEA extractors. The training set is also utilized for training the autoencoders. As the autoencoders require both training and validation sets, 80% of the training set is used as training examples, while the remaining 20% is used to validate the autoencoder. The validation portion of the dataset is used to find the optimal hyperparameters of the segmentation algorithms. After the best performing model configurations on the validation set are found, the test set is used to make the final comparisons between the different segmentation algorithms.

For a detailed presentation of the considered hyperparamters see Supplementary Tables S1, S2 in the Appendix. Furthermore, Supplementary Tables S3, S4 in the Appendix shows the hyperparameters selected for the offline and online evaluation respectively.

### 5.3 Evaluation metrics

The performance of the offline algorithms are evaluated based on the ScoreRegimes from Table 3 in Gharghabi et al. (2017). The definition of the ScoreRegimes is as follows:

$$\text{ ScoreRegimes }=\frac{\sum_{i=1}^{N_{GT}}\left|CP_{pred}-CP_{actual}\right|}{N_{GT}*n}$$ (5)

Where *N*_*GT*_ is the number of ground truth change-points, and *n* is the length of the time series.

Note that the ScoreRegimes requires knowing the number of ground truth change-points and are thus ill-adapted to evaluate online CPD algorithms as they might identify a different number of change-points compared to the ground truth. Thus, the online algorithms are evaluated using the Prediction Loss mean absolute error (MAE), which is a slight modification of Eq. 12 in Lee et al. (2018). The Prediction Loss MAE used in this work is defined as follows:

$$\text{ Prediction loss }MAE=\left|1-\frac{N_{\text{pred }}}{N_{GT}}\right|\times MAE$$ (6)

Where *N*_pred_ is the number of predicted change-points and *N*_*GT*_ is the number of ground truth change-points. Thus, instead of relying on pre-defined change-points, the Prediction loss MAE weights the mean absolute error with the prediction ratio. Importantly, as the metrics used for the offline and online case aren’t related, it is also not possible to make comparisons between the online and offline algorithm’s performance based on them.

As suggested in Demšar (2006), a two-step statistical procedure is applied to compare LS-USS against the relevant CPD algorithms. First, Friedman’s test ranks the algorithms amongst each other. Then, Holm’s post-hoc test is applied using the best ranked method as a comparison basis. The null hypothesis of the *post hoc* test is that the performance of the two models is the same. The null hypothesis is rejected when *p* < 0.05.

## 6 Results

The comparisons in this section are made from the five datasets in both the offline and online setting: UCI, EMG artificial, EMG, Dance Artificial and Dance.

### 6.1 Offline

In this subsection, for each dataset and participant, the full time series was available when performing unsupervised segmentation and the number of change-points in each time series was also known. Table 2 presents the results obtained in the offline setting.

**TABLE 2:** Comparison between the considered CPD algorithms in the offline setting.

UCI			
	**LS-USS**	FLUSS	LFMD
Mean Score Regimes	**0.00687**	0.00931	0.01580
Friedman Rank	**1.33**	2.07	2.60
H0 (Adjusted *p*-value)	**−**	0 (0.04461)	0 (0.00105)
Mean MAE	**164 (3.28** **s)**	213 (4.26 s)	369 (7.38 s)
EMG Artificial			
	**LS-USS**	FLUSS	LFMD
Mean Score Regimes	**0.00214**	0.00718	0.01839
Friedman Rank	**1.03**	1.97	3.00
H0 (Adjusted *p*-value)	**−**	0 (0.00030)	0 (<0.00001)
Mean MAE	**84 (0.08** **s)**	274 (0.27 s)	1,219 (1.22 s)
EMG			
	**LS-USS**	FLUSS	LFMD
Mean Score Regimes	**0.00452**	0.00593	0.00715
Friedman Rank	**1.00**	2.00	3.50
H0 (Adjusted *p*-value)	**−**	0 (0.01431)	0 (<0.00001)
Mean MAE	**917 (0.91** **s)**	1,200 (1.2 s)	2,449 (2.45 s)
Dance Artificial			
	LS-USS	**FLUSS**	LFMD
Score Regimes	0.01802	**0.01735**	0.03807
Friedman Rank	1.54	**1.54**	2.92
H0 (Adjusted *p*-value)	1	**−**	0 (<0.00001)
Mean MAE	158 (3.16 s)	**151 (3.02** **s)**	331 (6.60 s)
Dance			
	**LS-USS**	FLUSS	LFMD
Mean Score Regimes	**0.02899**	0.03806	0.03665
Friedman Rank	**1.47059**	2.47059	2.05882
H0 (Adjusted *p*-value)	**—**	0 (0.00710)	1
Mean MAE	**512 (10.24 s)**	637 (12.74 s)	629 (12.58 s)

For each dataset, values corresponding to the best performing algorithm are highlighted in bold.

### 6.2 Online

In this subsection, data was fed to the CPD algorithms as if they were acquired in real-time. Further, the total number of change-points in a time series was not given. Table 3 presents the results obtained in the online setting.

**TABLE 3:** Comparison between the considered CPD algorithms in the online setting. Note that the Prediction Loss MAE is calculated as the average value across the test set for each dataset.

UCI					
	***ɛ*** **LS-USS**	LS-USS Online	*ɛ* FLUSS	FLOSS	LFMD
Prediction Loss MAE	**45.54**	114.82	47.27	87.36	51.99
Friedman Rank	**2.50**	4.20	2.56	3.12	2.60
H0 (Adjusted *p*-value)	**-**	0 (0.01294)	1	1	1
Mean Prediction Ratio	**1.02**	1.49	1.03	0.93	1.11
Mean MAE	**326 (6.52 s)**	262 (5.24** **s)	304 (6.08** **s)	508 (10.16** **s)	316 (6.32** **s)
EMG Artificial					
	***ɛ*** **LS-USS**	LS-USS Online	*ɛ* FLUSS	FLOSS	LFMD
Prediction Loss MAE	**6.64**	16.10	26.73	86.44	97.54
Friedman Rank	**1.82**	2.28	2.80	3.88	4.22
H0 (Adjusted *p*-value)	**—**	1	0 (0.03202)	0 (<0.00001)	0 (<0.00001)
Mean Prediction Ratio	**1.00**	1.00	1.02	0.95	1.08
Mean MAE	**163 (0.16 s)**	258 (0.26** **s)	304 (0.30** **s)	626 (0.63** **s)	721 (0.72** **s)
EMG					
	***ɛ*** **LS-USS**	LS-USS Online	*ɛ* FLUSS	FLOSS	LFMD
Prediction Loss MAE	**121.97**	164.68	251.41	163.71	388.82
Friedman Rank	**1.50**	2.50	3.70	2.55	4.75
H0 (Adjusted *p*-value)	**—**	1	0 (0.00003)	1	0 (<0.00001)
Mean Prediction Ratio	**1.00**	0.94	0.87	0.98	0.81
Mean MAE	**1,430 (1.43 s)**	1,681 (1.68** **s)	1709 (1.71** **s)	1,621 (1.62** **s)	2,116 (2.12** **s)
Dance Artificial					
	***ɛ*** **LS-USS**	LS-USS Online	*ɛ* FLUSS	FLOSS	LFMD
Prediction Loss MAE	**27.15**	64.07	48.00	55.05	109.04
Friedman Rank	**1.92**	3.17	2.77	2.80	4.34
H0 (Adjusted *p*-value)	**—**	0 (0.00023)	0 (0.01078)	0 (0.01078)	0 (0.00000)
Mean Prediction Ratio	**0.91**	0.83	0.85	0.87	1.86
Mean MAE	**177 (3.54 s)**	260 (5.20** **s)	235 (4.70** **s)	272 (5.44** **s)	129 (2.58** **s)
Dance					
	*ɛ* LS-USS	**LS-USS Online**	*ɛ* FLUSS	FLOSS	LFMD
Prediction Loss MAE	415.31	**204.12**	281.01	334.65	289.20
Friedman Rank	3.88	**2.17**	2.71	3.12	3.12
H0 (Adjusted *p*-value)	1	**-**	1	1	1
Mean Prediction Ratio	0.83	**1.18**	1.26	0.94	1.25
Mean MAE	785 (15.7 s)	**411 (8.22 s)**	397 (7.94** **s)	593 (11.86** **s)	468 (9.36** **s)

For each dataset, values corresponding to the best performing algorithm are highlighted in bold.

## 7 Discussion

### 7.1 Observations from the hyperparameter search

The results presented in Supplementary Table S1 in the appendix shows that LREA is chosen 12 out of 15 times across the five datasets for the three algorithms considered in the offline setting. This indicates that scaling the CAC based on the local statistics is useful when dealing with longer time series data. Another interesting finding from the hyperparameter selection is that the autoencoder selected by the hyperparameter search for the LS-USS models (both offline and online) varies between the datasets; some use the less complex, fully connected model, while others use the convolutional model. As alluded to in Lee et al. (2018), using a smaller model with only two hidden layers might be beneficial for feature representation as it could lead to a more general way to represent the data. The drawback of simpler autoencoder models is that one risks losing some of the information needed for segment differentiation. Note that an in-depth analysis of the impact of the autoencoder’s architecture was outside the scope of the current work and will be the focus of future works.

### 7.2 Offline

Table 2 illustrates that LS-USS outperforms the other segmentation algorithms significantly and consistently on three of the five datasets considered in the offline setting, namely, UCI, EMG, and EMG artificial. On the Dance Artificial dataset, LS-USS and FLUSS exhibit comparable performance, while LFMD performs significantly worst. Contrastingly, while LS-USS significantly outperforms FLUSS on the Dance dataset, there is no significant difference between it and LFMD (despite achieving a higher ranking than LFMD). These results suggest that incorporating the multidimensionality of time series data by learning a specific representation provides a clear advantage compared to using FLUSS directly. Additionally, the poor performance of LFMD highlights the utility of leveraging the latent space matrix profile for time series segmentation.

Overall, LS-USS demonstrates good performance on the offline datasets, as evident from the Mean ScoreRegimes and Mean MAE metrics. However, a more thorough evaluation of its performance can be done by considering the time scale of the datasets. For instance, the UCI data typically consists of segments that are 20–60 s long, and LS-USS is off by an average of 3.22 s compared to 4.26 s for FLUSS and 7.38 s for LFMD. For the EMG datasets, which have a segment length of around 5 s, LS-USS is off by 0.08 s and 0.91 s on average for the EMG artificial and EMG datasets respectively. The dance dataset, on the other hand, has segment lengths of around 20 s long, with LS-USS detecting change-points that are 3.16 s off on average for the artificial dataset and 10.24 s off for the non-artificial dataset. It’s important to note that not all change-points are perfectly matched to their actual locations, which may result in some false positives. This could have significant impact on the MAE metric, especially for longer time series, if the algorithm detects too many false positives on non-trivial data. To address this issue, the ScoreRegimes evaluation metric normalizes the score between 0 and 1 based on the number of segments and the length of the time series, which as discussed in Section 5.3 makes it a more robust metric for offline CPD. Furthermore, it’s worth keeping in mind that the change-points are defined by humans, who may not be entirely precise. As a result, some discrepancies between the ground truth and the LS-USS predictions may not necessarily be indicative of poor algorithmic performance. Overall, LS-USS shows promising results, but it’s important to consider these factors when interpreting the metrics.

### 7.3 Online

The UCI dataset results reveal that LS-USS is the top-ranked algorithm, but its performance doesn’t differ significantly from that of other algorithms. However, when tested on both the EMG and EMG Artificial datasets, *ɛ*-real time LS-USS significantly outperforms all other algorithms, except for LS-USS online on the EMG Artificial dataset and LS-USS online and FLOSS on the EMG dataset. In addition, when evaluated on the Dance Artificial dataset, *ɛ*-real time LS-USS once again shows significant improvement over the other algorithms. However, when tested on the Dance dataset, the results are inconsistent, with LS-USS Online outperforming all other models, while *ɛ*-LS-USS ranks last among all the tested algorithms. Statistical analysis confirms that there is significant variation in the performance of algorithms between recordings, as none of the algorithms is significantly better than any other. This is likely due to their sensitivity to the threshold parameters which might not generalize well between the training and test set for the dance dataset (and also between the recordings in the test set itself). Overall, the *ɛ*-real time LS-USS LTEA model performs the best across all datasets as an online segmentation algorithm.

Upon examining the real-world performance of the *ɛ*-real time LS-USS algorithm on online datasets, it is interesting to observe its performance compare to the offline setting. For example, the *ɛ*-real time LS-USS algorithm is on average 6.25 s off on the UCI dataset compared to 3.28 s when offline, 1.43 s off on the EMG dataset (0.91 s offline), and 0.63 s off on the EMG artificial dataset (0.08 s offline). Additionally, the performance of the LS-USS algorithm on the dance dataset is also comparable to its online counterpart. Overall, amongst the online algorithms, he results also shows that LS-USS online tends to perform slightly worse than *ɛ*-real time LS-USS, likely due to the limitation of considering only right pointing arcs. This pattern is also observed between the FLUSS and FLOSS algorithms. As previously mentioned in Section 5.3, it is important to take into account that comparing online algorithms solely on the basis of MAE can be deceiving, as the algorithms must predict not only the change-points but also the number of change-points. This issue is exemplified by the performance of LFMD on the Dance Artificial dataset, where it achieved the best MAE score but was ranked the lowest overall. The algorithm tended to over-predict the number of change-points, resulting in a mean prediction ratio of 1.86. While this resulted in some inaccuracies, it also led to a higher density of predicted change-points, bringing at least some of them closer to the ground-truth values. As a result, the MAE score improved, giving a false impression of a well performing algorithm if looking at the MAE metric alone. This emphasizes the importance of weighing the prediction rate with distance metric MAE, as seen in the evaluation metric Prediction loss MAE.

**FIGURE 11:**
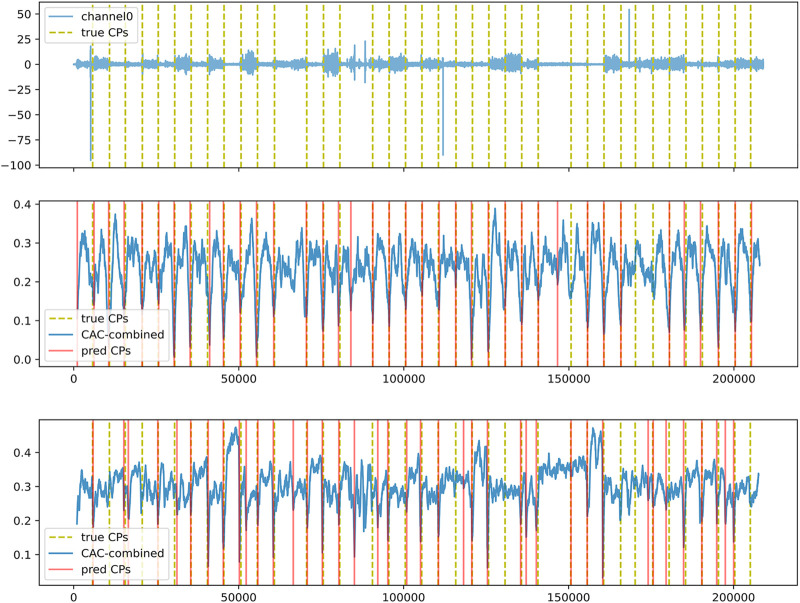
Top: Channel 0 of participant3_evaluation3 in the EMG dataset. Middle: Segmentation using LS-USS–LREA- Bottom: Segmentation using LS-USS–LTEA.

Overall, and as anticipated, the offline setting proved to be easier in comparison to the online setting, owing to its access to the true number of change-points and full time series data for making decisions. In contrast, the online setting posed a challenge as the number of change-points was unknown and their identification solely relied on past information. Despite this disparity, the difference in performance wasn’t substantial, indicating the effectiveness of the threshold-based approach proposed with LTEA in identifying significant change-points in an online setting. Notably, *ɛ*-real time LS-USS demonstrated an average prediction rate ranging from 0.83 to 1.02 across multiple datasets, underscoring the reliability of LTEA in extracting change-points from the CAC. Figure 11 shows how the LTEA extractor compares to the LREA extractor on a typical time series of the EMG dataset. For a more detailed understanding of the performance of LS-USS in both offline and online settings for each dataset, we provide visually illustrative examples in Section 2 of the appendix.

### 7.4 Limitations

This work’s main limitation is that the experiments conducted have mainly been on data with similar sampling rates, and numerical data only. Therefore, how the algorithm performs when considering time series data containing channels with both numerical and categorical variables remains to be evaluated. Furthermore, doing an in-depth analysis on how the number of channels affects the performance of LS-USS is also required and will be conducted in future works. Additionally, future works will also consider other type of model to learn the latent space such as a variational autoencoder.

The performance of the segmentation algorithms is heavily influenced by the hyperparameter search process. Therefore, a more comprehensive hyperparameter search that includes sampling from predefined distributions should be conducted instead of relying on a simple grid search. Moreover, evaluating the sensitivity of hyperparameter selection is crucial to determine which hyperparameters require more thorough optimization and to identify any potential hyperparameters that generalize well using a constant value. This process helps us understand which hyperparameters have a significant impact on the algorithm’s performance and which ones can be set to a constant value without affecting the results significantly. Identifying such hyperparameters can streamline the optimization process and make the algorithm more practical and efficient in real-world scenarios. Additionally, future research should explore alternative approaches to segmentation based on the LSMP. A promising starting point would be to investigate the application of techniques used in Rodrigues et al. (2022), such as novelty search, periodic search, and similarity profile, to the LSMP instead of the feature-based self-similarity matrix.

## 8 Conclusion

The main contribution of this work are the segmentation algorithms LS-USS and LS-USS online. Through extensive testing conducted on both artificial and real-world datasets from various domains and sensors, it was found that LS-USS generally delivers on par or better segmentation scores compared to other state-of-the-art algorithms such as FLUSS/FLOSS and LFMD. The LS-USS algorithms have many desirable properties. They can be implemented both online and in an “anytime” fashion. They are domain agnostic (beyond knowing the order of time scale considered for change-points) and do not need extensive tuning of hyperparameters to achieve state-of-the-art performance. Further, they don’t make any statistical assumptions about the input data. The LSMP component used in LS-USS also shows that it is possible to calculate a temporarily constrained matrix profile from feature vectors by exploiting highly parallelized hardware. The same methods used for constructing the LSMP can be used on any vector-based Representation Learning algorithms Bengio et al. (2013), which represents an excellent potential direction for future research.

The extraction algorithms LREA and LTEA presented in this work also show potential. LREA usually outperformed the more global REA algorithm, which shows that scaling the CAC based on the local statistics is highly useful, especially for long time series data. The online extraction algorithm LTEA uses the same CAC scaling as LREA but uses a threshold to identify the change-points instead of extracting the *n* lowest “valley” points. Consequently, in contrast to REA and LREA, LTEA doesn’t need any information on the number of change-points to extract, making it applicable to a wider array of real-world segmentation problems.

## Data Availability

Publicly available datasets were analyzed in this study. This data can be found here: The UCR Dataset (https://www.cs.ucr.edu/%7Eeamonn/time_series_data_2018), the UCI Dataset (https://archive.ics.uci.edu/ml/datasets/human+activity+recognition+using+smartphones), the Long Term EMG Dataset (https://github.com/UlysseCoteAllard/LongTermEMG) and the Dance Dataset (https://doi.org/10.1145/3323213). Further, the modified datasets used in this work, notably the EMG Artificial and Dance Artificial are available at the following link: https://github.com/UlysseCoteAllard/SemanticSegmentationDatasets.
