# Supplementary material for: Latent space unsupervised semantic segmentation
Source: Front Physiol. 2023 Apr 25;14:1151312. doi: 10.3389/fphys.2023.1151312 (PMC10166858; doi:10.3389/fphys.2023.1151312)
Supplement: Supplementary file 1 [file DataSheet1.pdf]

Latent Space Unsupervised Semantic Segmentation - Appendix

1 HYPERPARAMETERS SEARCH AND SELECTION

Table S1 and Table S2 show the hyperparameters considered for the offline and online algorithms respectively.

**Offline Models**	**Data Scalers**	**NW**		**TC**		**Step-Size**		**Autoencoder**	**Extraction Algorithm**
	All Datasets	All datasets		EMG Datasets	UCI	All datasets		All Datasets	All Datasets
LS-USS	No Scaler<br>Standard Scaler<br>Robust Scaler<br>Min Max Scaler	50<br>100<br>150<br>200	300<br>400<br>500	1000<br>1500<br>2000	800<br>1200<br>1600	-	-	Fully Connected<br>Convolutional	REA<br>LREA
FLUSS	No Scaler<br>Standard Scaler<br>Robust Scaler<br>Min Max Scaler	50<br>100<br>150<br>200	300<br>400<br>500	1000<br>1500<br>2000	800<br>1200<br>1600	-	-	-	REA<br>LREA
LFMD	No Scaler<br>Standard Scaler<br>Robust Scaler<br>Min Max Scaler	50<br>100<br>150<br>200	300<br>400<br>500	-	-	25<br>50<br>100<br>150	200<br>250<br>350<br>500	Fully Connected<br>Convolutional	REA<br>LREA

Table S1. Hyperparameters considered for the CPD algorithms in the offline setting. NW is the sub-sequence length. TC is the temporal constraint.

**Online Models**	**Data Scalers**	**NW**		**TC**		**Step-size**		**Autoencoder**	**NW**		**Extraction Algorithm**
	All Datasets	All datasets		EMG datasets	UCI	All datasets		All Datasets	All datasets		All Datasets
LS-USS<br>(ε-real time)	No Scaler<br>Standard Scaler<br>Robust Scaler<br>Min Max Scaler	50<br>100<br>150<br>200	300<br>400<br>500	1000<br>1500<br>2000	800<br>1200<br>1600	-	-	Fully Connected<br>Convolutional	-0.5<br>-1.0<br>-1.5	-2.0<br>-2.5<br>-3.0	LTEA
LS-USS - Online	No Scaler<br>Standard Scaler<br>Robust Scaler<br>Min Max Scaler	50<br>100<br>150<br>200	300<br>400<br>500	1000<br>1500<br>2000	800<br>1200<br>1600	-	-	Fully Connected<br>Convolutional	-0.5<br>-1.0<br>-1.5	-2.0<br>-2.5<br>-3.0	LTEA
FLUSS<br>(ε-real time)	No Scaler<br>Standard Scaler<br>Robust Scaler<br>Min Max Scaler	50<br>100<br>150<br>200	300<br>400<br>500	1000<br>1500<br>2000	800<br>1200<br>1600	-	-	-	-0.5<br>-1.0<br>-1.5	-2.0<br>-2.5<br>-3.0	LTEA
FLOSS	No Scaler<br>Standard Scaler<br>Robust Scaler<br>Min Max Scaler	50<br>100<br>150<br>200	300<br>400<br>500	1000<br>1500<br>2000	800<br>1200<br>1600	-	-	-	-0.5<br>-1.0<br>-1.5	-2.0<br>-2.5<br>-3.0	LTEA
LFMD	No Scaler<br>Standard Scaler<br>Robust Scaler<br>Min Max Scaler	50<br>100<br>150<br>200	300<br>400<br>500	-	-	25<br>50<br>100<br>150	200<br>250<br>350<br>500	Fully Connected<br>Convolutional	-0.5<br>-1.0<br>-1.5	-2.0<br>-2.5<br>-3.0	LTEA

Table S2. Hyperparameters considered for the CPD algorithms in the online setting. NW is the sub-sequence length. TC is the temporal constraint. The threshold parameter is the threshold used in the LTEA extraction algorithm.

Tables S3 and S4 shows the hyperparameters selected for the offline and online algorithms after doing hyperparameter search. The selected parameters is the ones used for the comparisons between the different models in Section VI and VII.

**UCI**						
**CPD Algorithm**	**Extraction Algorithm**	**Data Scaler**	**NW**	**TC**	**Step-size**	**Autoencoder**
LFMD	LREA	Robust Scaler	50	-	250	Fully Connected
LS-USS	REA	Min Max Scaler	50	800	1	Fully Connected
FLUSS	LREA	Robust Scaler	50	1200	1	-
**EMG Artificial**						
**CPD Algorithm**	**Extraction Algorithm**	**Data Scaler**	**NW**	**TC**	**Step-size**	**Autoencoder**
LFMD	REA	Standard Scaler	200	-	500	Conv. Model
LS-USS	LREA	Standard Scaler	50	1500	1	Conv. Model
FLUSS	LREA	Standard Scaler	50	1500	1	-
**EMG**						
**CPD Algorithm**	**Extraction Algorithm**	**Data Scaler**	**NW**	**TC**	**Step-size**	**Autoencoder**
LFMD	LREA	Standard Scaler	50	-	100	Fully Connected
LS-USS	LREA	Robust Scaler	50	2000	1	Conv. Model
FLUSS	LREA	Standard Scaler	50	1500	1	-
**Dance Artificial**						
**CPD Algorithm**	**Extraction Algorithm**	**Data Scaler**	**NW**	**TC**	**Step-size**	**Autoencoder**
LFMD	LREA	Min Max Scaler	100	-	100	Fully Connected
LS-USS	LREA	No Scaler	50	800	1	Conv. Model
FLUSS	LREA	Standard Scaler	50	800	1	-
**Dance**						
**CPD Algorithm**	**Extraction Algorithm**	**Data Scaler**	**NW**	**TC**	**Step-size**	**Autoencoder**
LFMD	LREA	Min Max Scaler	400	-	100	Fully Connected
LS-USS	LREA	Min Max Scaler	400	1600	1	Fully Connected
FLUSS	REA	Robust Scaler	400	800	1	-

Table S3. Hyperparameters selected for the compared CPD algorithms in the offline setting. NW is the sub-sequence length. TC is the temporal constraint.

2 TIME SERIES SEGMENTATION EXAMPLES

UCI

CPD Algorithm	Extraction Algorithm	Data Scaler	NW	TC	Step-size	Autoencoder	Threshold
LFMD	LTEA	Min Max Scaler	100	-	100	Fully Connected	-2
FLUSS	LTEA	Robust Scaler	50	1600	1	-	-1
FLOSS	LTEA	Robust Scaler	150	1600	1	-	-1
LS-USS	LTEA	Min Max Scaler	150	800	1	Fully Connected	-1.5
LS-USS Online	LTEA	Robust Scaler	50	1200	1	Fully Connected	-0.5

EMG Artificial

CPD Algorithm	Extraction Algorithm	Data Scaler	NW	TC	Step-size	Autoencoder	Threshold
LFMD	LTEA	Standard Scaler	300	-	100	Fully Connected	-0.5
FLUSS	LTEA	Standard Scaler	50	1500	1	-	-1
FLOSS	LTEA	Standard Scaler	100	2000	1	-	-0.5
LS-USS	LTEA	Standard Scaler	50	2000	1	Conv. Model	-0.5
LS-USS Online	LTEA	Standard Scaler	50	1500	1	Conv. Model	-1

EMG

CPD Algorithm	Extraction Algorithm	Data Scaler	NW	TC	Step-size	Autoencoder	Threshold
LFMD	LTEA	Standard Scaler	500	-	500	Fully Connected	-1.5
FLUSS	LTEA	Standard Scaler	50	1000	1	-	-2
FLOSS	LTEA	Standard Scaler	200	1000	1	-	-2
LS-USS	LTEA	Standard Scaler	300	1000	1	Fully Connected	-2
LS-USS Online	LTEA	Standard Scaler	200	1500	1	Fully Connected	-2

Dance Artificial

CPD Algorithm	Extraction Algorithm	Data Scaler	NW	TC	Step-size	Autoencoder	Threshold
LFMD	LTEA	Min Max Scaler	50	-	50	Fully Connected	-0.5
FLUSS	LTEA	Min Max Scaler	100	800	1	-	-0.5
FLOSS	LTEA	No Scaler	50	800	1	-	-0.5
LS-USS	LTEA	Min Max Scaler	50	800	1	Conv. Model	-0.5
LS-USS Online	LTEA	Min Max Scaler	50	800	1	Conv. Model	-0.5

Dance

CPD Algorithm	Extraction Algorithm	Data Scaler	NW	TC	Step-size	Autoencoder	Threshold
LFMD	LTEA	Min Max Scaler	125	-	200	Fully Connected	-1.5
FLUSS	LTEA	Robust Scaler	200	1200	1	-	-1.5
FLOSS	LTEA	Robust Scaler	125	1200	1	-	-2
LS-USS	LTEA	Roboust Scaler	200	1200	1	Fully Connected	-2
LS-USS Online	LTEA	Min Max Scaler	200	1600	1	Fully Connected	-1.5

Table S4. Hyperparameters selected for the compared CPD algorithms in the online setting. NW is the sub-sequence length. TC is the temporal constraint. The threshold parameter is the threshold used in the LTEA extraction algorithm.

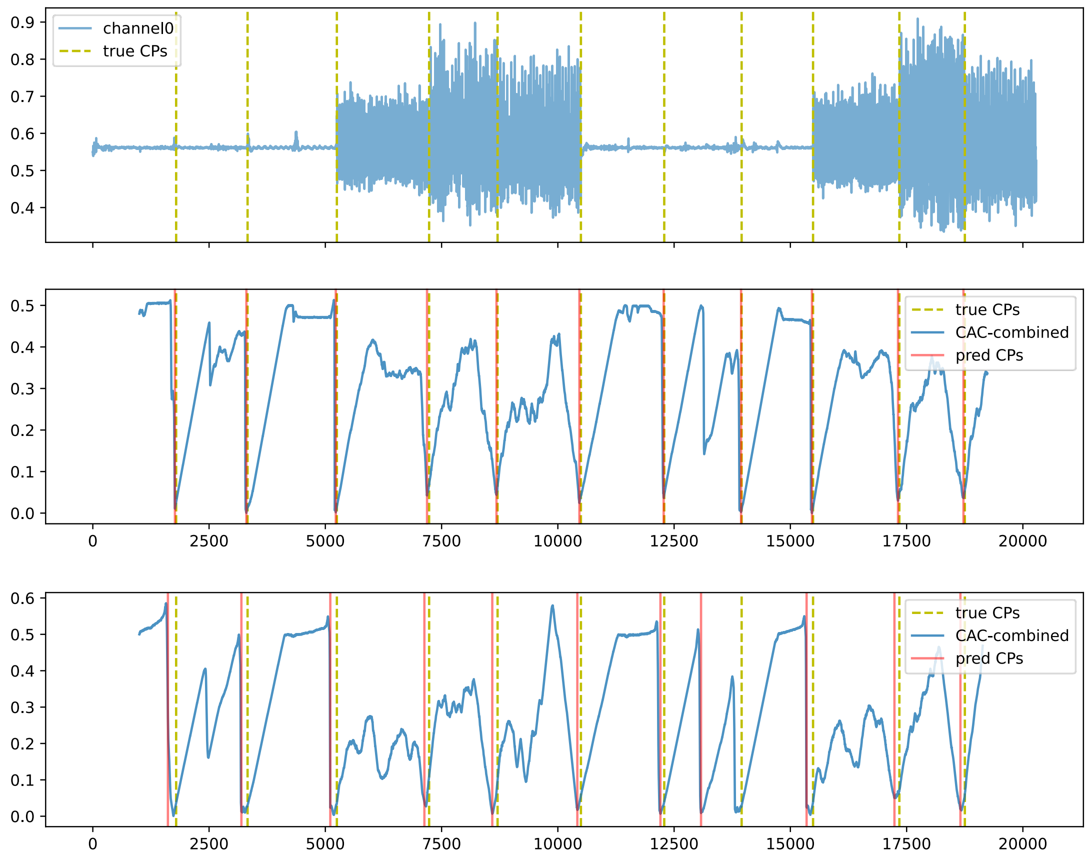

Figure S1. Top: Channel 0 of subject 4 in the UCI dataset. Middle: Segmentation using LS-USS – LREA-Bottom: Segmentation using LS-USS – LTEA

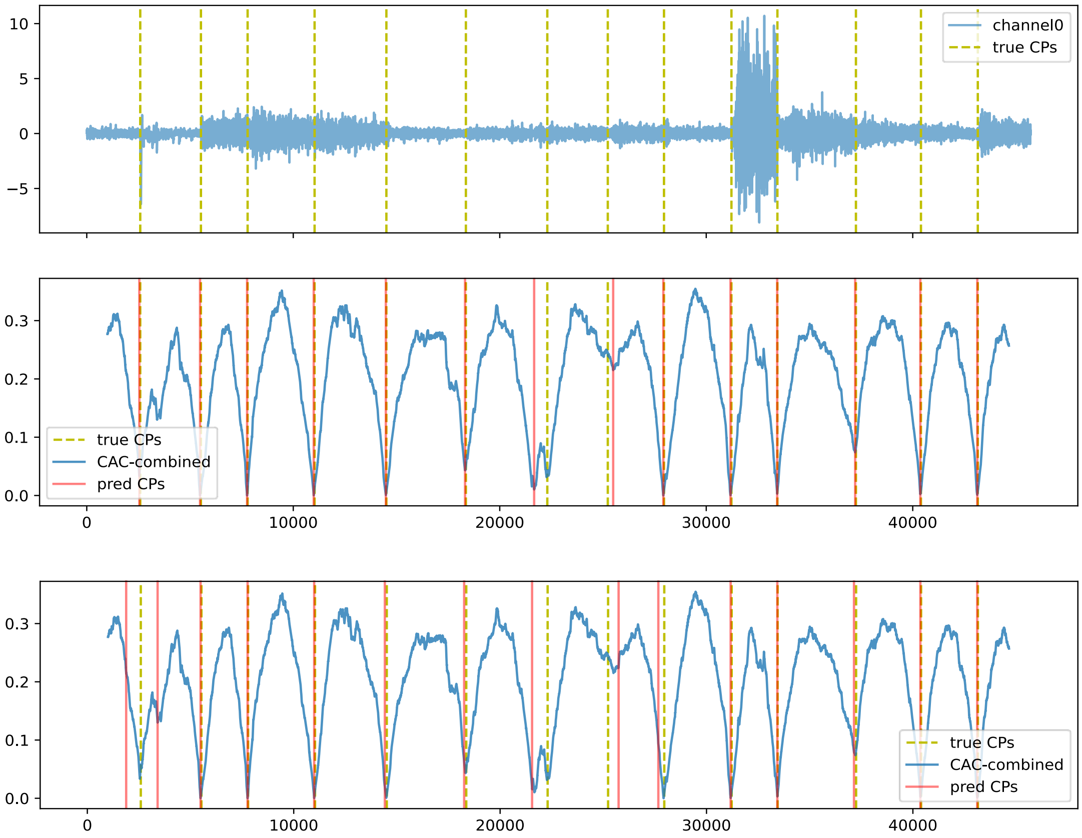

Figure S2. Top: Channel 0 of gesture19 in the EMG Artificial dataset. Middle: Segmentation using LS-USS – LREA- Bottom: Segmentation using LS-USS – LTEA.

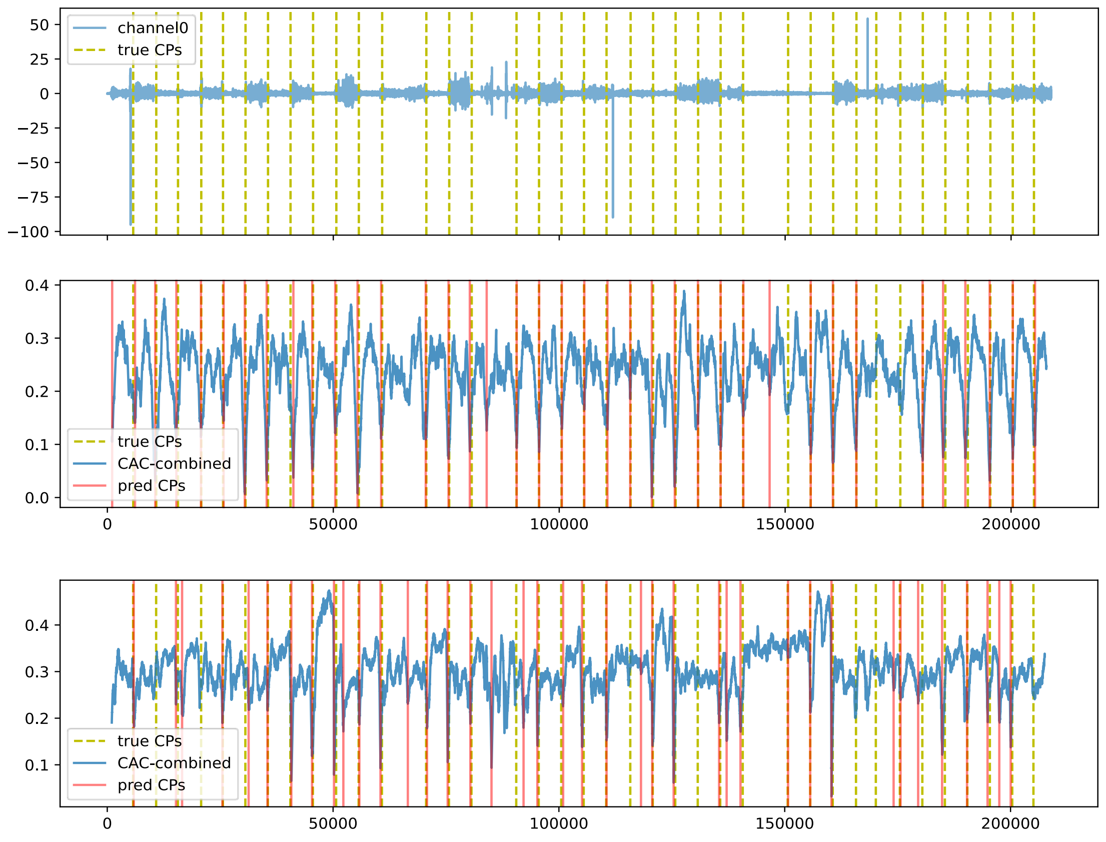

Figure S3. Top: Channel 0 of participant3_evaluation3 in the EMG dataset. Middle: Segmentation using LS-USS – LREA- Bottom: Segmentation using LS-USS – LTEA.

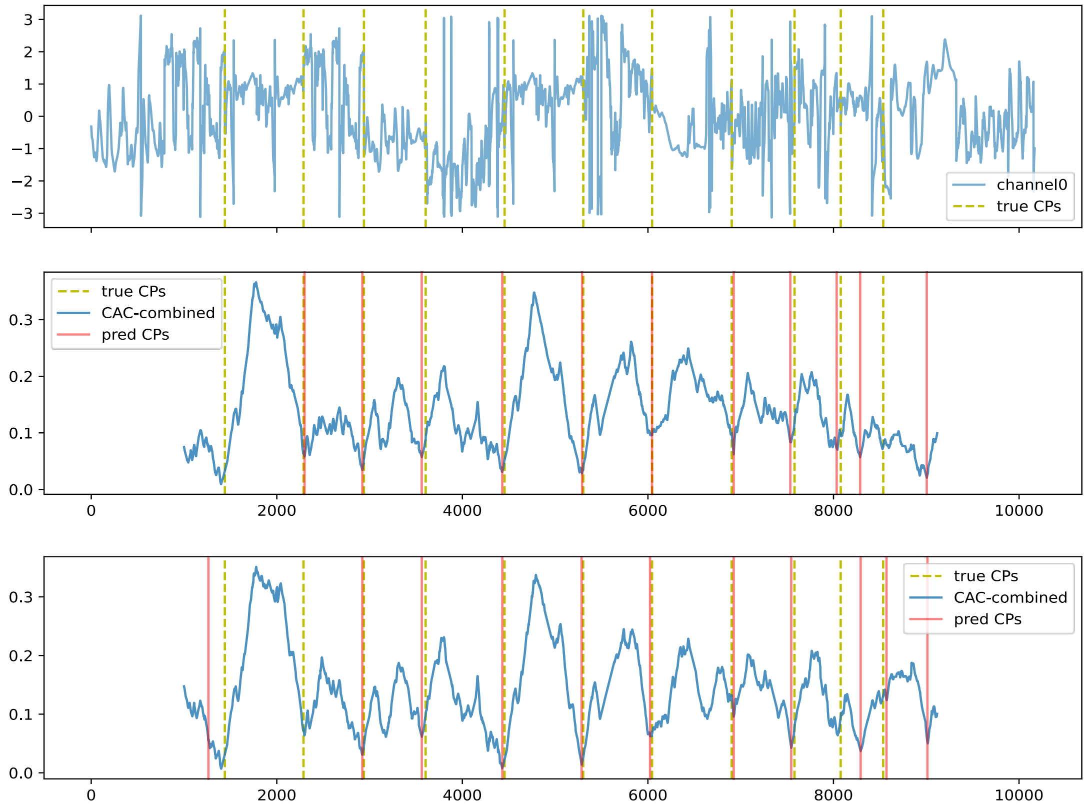

Figure S4. Channel 0 of d41 in the Dance Artificial dataset. Middle: Segmentation using LS-USS – LREA- Bottom: Segmentation using LS-USS – LTEA.

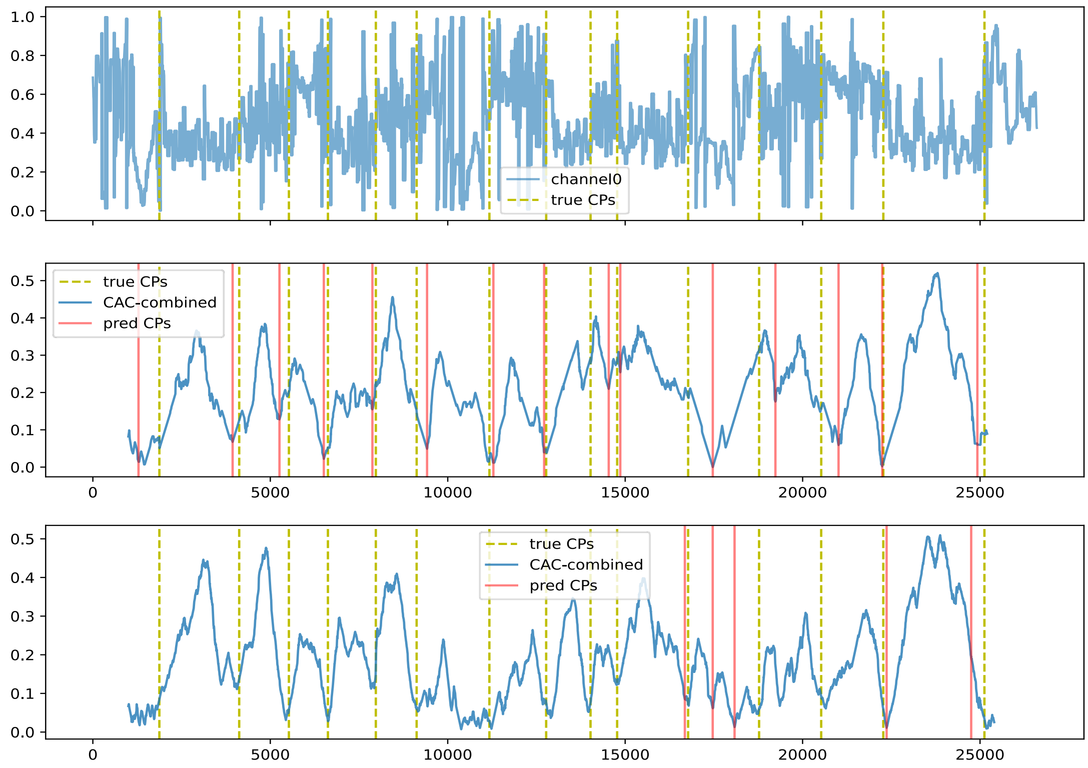

Figure S5. Top: Channel 0 of d24 in the Dance dataset. Middle: Segmentation using LS-USS – LREA- Bottom: Segmentation using LS-USS – LTEA (shows the effects of setting the threshold parameter to high).
